# The combination of tamoxifen with amphotericin B, but not with fluconazole, has synergistic activity against the majority of clinical isolates of *Cryptococcus neoformans*

**DOI:** 10.1111/myc.12955

**Published:** 2019-06-23

**Authors:** Trieu Phan Hai, Anh Duong Van, Nguyen Thi Thuy Ngan, Le Thanh Hoang Nhat, Nguyen Phu Huong Lan, Nguyen V. Vinh Chau, Guy E. Thwaites, Damian Krysan, Jeremy N. Day

**Affiliations:** ^1^ Oxford University Clinical Research Unit Ho Chi Minh City Viet Nam; ^2^ Cho Ray Hospital Ho Chi Minh city Viet Nam; ^3^ Hospital for Tropical Diseases Ho Chi Minh city Viet Nam; ^4^ Centre for Tropical Medicine, Nuffield Department of Medicine University of Oxford Oxford UK; ^5^ Department of Pediatrics and Microbiology/Immunology University of Iowa Iowa City Iowa USA

**Keywords:** antifungal, cryptococcal meningitis, *Cryptococcus neoformans*, drug repurposing, susceptibility, synergy, tamoxifen, treatment

## Abstract

**Background:**

Cryptococcal meningitis has fatality rates of 40%‐70%, resulting in 200 000 deaths each year. The best outcomes are achieved with amphotericin combined with flucytosine but flucytosine is expensive and unavailable where most disease occurs. More effective and affordable treatments are needed. Tamoxifen, a selective oestrogen receptor modulator frequently indicated for breast cancer, has been found to have synergistic activity against the *Cryptococcus neoformans* type strain when combined with amphotericin or fluconazole. It is cheap, off‐licence, widely available and well‐tolerated, and thus a pragmatic potential treatment for cryptococcal disease.

**Objectives:**

We wanted to determine the susceptibility of clinical isolates of *C. neoformans* to tamoxifen alone and in combination with other antifungals, to determine whether there is sufficient evidence of activity to justify a clinical trial.

**Methods:**

We used the CLSI broth microdilution protocol to test the susceptibility of 30 randomly selected clinical isolates of *C. neoformans* to tamoxifen, in dual combination with amphotericin, fluconazole or flucytosine, and in triple combination with amphotericin and fluconazole. Evidence of drug interactions was assessed using the fractional inhibitory concentration index.

**Results:**

The MIC50 and MIC90 of tamoxifen were 4 and 16 mg/L, respectively. The combination of tamoxifen and amphotericin suggested a synergistic interaction in 20 of 30 (67%) isolates. There was no interaction between tamoxifen and either fluconazole or flucytosine. Synergy was maintained in 3‐Dimensional chequerboard testing. There was no evidence of antagonism**.**

**Conclusions:**

Tamoxifen may be a useful addition to treatment with amphotericin and fluconazole for cryptococcal meningitis; a trial is justified.

## 1 INTRODUCTION

Every year, more than 200 000 people die from cryptococcal meningitis.^1^ The vast majority of cases are due to *Cryptococcus neoformans* and occur in patients who have underlying immunosuppression. Currently, the most frequent cause of immunosuppression is HIV infection, but iatrogenic causes, including therapy for connective tissue disorders, cancers and solid organ transplantation, are increasingly important as healthcare advances.^2^ Cryptococcal meningitis also occurs in immunocompetent patients, where *Cryptococcus gattii sensu lato* is an important cause, particularly in the tropics, Western Canada and the Pacific Northwest of the USA.^2^ In Southeast and East Asia, both *C. neoformans* and *C. gattii sensu lato* cause disease in apparently immunocompetent patients.^3^

Treatment guidelines for cryptococcal meningitis are based upon the results of a number of randomised controlled trials, largely completed in patients with HIV infection.^4^, ^5^, ^6^, ^7^, ^8^ The best clinical outcomes are achieved using induction treatment consisting of amphotericin combined with flucytosine.^6^, ^9^ This combination was first shown to deliver improved rates of sterilisation of cerebrospinal fluid (CSF) compared with amphotericin monotherapy in 1997; subsequently, a trial from Vietnam demonstrated that as well as delivering faster rates of clearance of yeast from CSF, it also resulted in better survival, with a 40% reduction in the risk of death by 10 weeks.^4^, ^6^ However, even on gold standard therapy, death rates 3 months after diagnosis remain high, at between 15 (USA) and 40% (Asia and Africa).^1^

Recently, the key role of flucytosine in delivering the best outcomes has been underlined by the publication of the ACTA trial.^9^ This study confirmed the role of amphotericin combined with flucytosine as the treatment of choice and also demonstrated that an oral regimen consisting of flucytosine combined with fluconazole delivered survival rates approaching that of amphotericin combined with flucytosine. However, despite having been off‐patent for several decades, and the existence of an energetic and passionate advocacy campaign, there has been little progress in improving access to flucytosine.^10^ In fact, prices have risen significantly in recent years.^11^

Flucytosine is an unattractive prospect for generic manufacturers because of its limited indications outside cryptococcal meningitis, and because the vast burden of cryptococcal disease is in low‐income countries. Both these factors reduce the possibility of generating significant profit, and this conundrum exists for any treatment specific for cryptococcal meningitis. The perfect drug for cryptococcal meningitis would be off‐patent, manufactured by a number of different companies, and frequently indicated for some other, common, disease. These circumstances would confer commercial viability driven by high numbers of prescriptions rather than high per tablet profit margins, keep prices affordable through competition and increase the likelihood of licensing and availability where cryptococcal disease occurs. This paradigm has driven interest in the repurposing of generic drugs for neglected tropical and other diseases of poverty.

Tamoxifen, a selective oestrogen reuptake modulator, was first noted to have antifungal activity against *Saccharomyces cerevisiae * in 1989.^12^ Soon afterwards, it was found to have antifungal action against *Candida albicans*, and against *C. neoformans* in 2009.^13^, ^14^ Tamoxifen has a wide range of effects in mammalian cells including anti‐oxidant activity, alteration of cell membrane properties and induction of apoptosis.^14^ In *Cryptococcus*, it has been shown to bind to calmodulin and a calmodulin‐like protein, preventing the activation of calcineurin, which is involved in the yeast stress response.^14^, ^15^ Furthermore, when tested against the *C. neoformans* type strain H99, tamoxifen interacted synergistically in combination with both amphotericin and fluconazole, and had a fungicidal effect in combination with fluconazole in the mouse infection model.^15^ Tamoxifen has high oral bioavailability, is lipophilic resulting in high brain concentrations, and is concentrated in macrophage phagosomes—a site of growth of *C. neoformans*.^15^, ^16^, ^17^ Therefore, tamoxifen, which is off‐patent, widely available and affordable, is a promising treatment to augment current antifungal therapy for cryptococcal meningitis. As a first step, we tested the susceptibility of clinical isolates of *C. neoformans* from our hospital in Vietnam to tamoxifen, amphotericin B, fluconazole and flucytosine, alone and in combination. Because we occasionally see disease in our patients due to *C. gattii sensu lato*, we also measured the susceptibility of a small number of isolates and control strains of these species to tamoxifen alone and in combination with amphotericin.

## 2 MATERIALS AND METHODS

### 2.1 Fungal isolates

We randomly selected thirty isolates of *C. neoformans* from the strain collection at our institute. The strain collection consists of isolates derived from the cerebrospinal fluid of patients at the point of diagnosis of cryptococcal meningitis. The patients were all enrolled into randomised controlled trials or prospective descriptive studies of cryptococcal meningitis (N = 299).^3^, ^6^ All 30 isolates were subjected to amphotericin and tamoxifen susceptibility testing; because testing revealed that evidence of drug interaction was rare between tamoxifen and either fluconazole or flucytosine, susceptibility testing to these drugs was limited to a subset of 20 isolates.

We also determined the susceptibility of four *C. gattii sl* isolates, derived from participants from the descriptive study of cryptococcal meningitis to amphotericin and tamoxifen.^3^ These consisted of three *C. gattii* and one *Cryptococcus deuterogatii*. We have not seen infection due to other members of the species complex in our hospital. For reference and quality control, we used the *C. neoformans* H99 type strain, *Candida krusei* (ATCC 6258), and four *C. gattii* strains representing the major molecular groups: *C. gattii sensu stricto* (WM179), *Cryptococcus deuterogattii* (WM178), *Cryptococcus bacillisporus* (WM175) and *Cryptococcus tetragattii* (WM779), kindly provided by Dr Wieland Meyer of Westmead Millenium Centre, Sydney Australia.

### 2.2 In vitro antifungal susceptibility testing

All antifungal drugs were obtained as pure drug from Sigma‐Aldrich, Germany. Fluconazole and flucytosine were prepared as stock solutions in sterile water. Amphotericin B (Sigma) and Tamoxifen were prepared in dimethyl sulfoxide (DMSO).

The minimum inhibitory concentration (MIC) of each of amphotericin and tamoxifen were determined according to the M27‐A3 broth microdilution protocol of the Clinical and Laboratory Standards Institute (CLSI).^18^ Briefly, antifungal agents and inoculum were prepared in RPMI 1640 (Difco) buffered to a pH of 7.0 using 0.165 mol/L morpholine propane sulphonic acid (MOPS; Sigma). The infectious inoculum—0.5‐2.5 × 10^3^ CFU/mL—was achieved using an automated cell counter Cellometer X2 (Nexcelom Bioscience) and serial dilution. The densities of all inocula were confirmed through culture. Serial twofold drug dilutions were prepared on 96‐well microtitre plates at the following concentrations: Fluconazole 0.125‐64 μg/mL, amphotericin 0.0625‐32 µg/mL, tamoxifen, 0.125‐64 µg/mL and flucytosine 0.125‐64 µg/mL. All microplates were incubated at 35°C for 72 hours. All plates were inspected by two observers, and the MIC estimated as the lowest concentration that completely inhibited visual growth for amphotericin and tamoxifen. For fluconazole and flucytosine, the endpoint was defined as the lowest drug concentration that resulted in an 80% reduction of visual growth compared with that of the drug‐free growth control well.

### 2.3 Chequerboard microdilution assays

Combinations of tamoxifen plus amphotericin, tamoxifen plus fluconazole, tamoxifen plus flucytosine and tamoxifen plus amphotericin plus fluconazole were tested as has been described previously.^19^ For two‐dimensional microplate preparation, a stock solution of each antifungal drug was made in RPMI at fourfold the final desired test concentrations. To test a combination of drug “A” with drug “B,” 50 µL of drug “A” solution at fourfold the desired final concentration was combined with 50 µL of the chosen combination drug “B” at fourfold its desired final concentration to give a volume of 100 µL. One hundred µL of inoculum was added to this to give a final volume of 200 µL (and hence a fourfold dilution of the original stock drug solution). The final drug concentrations tested in combination were as follows: tamoxifen from 0.25 to 64 µg/mL; fluconazole and flucytosine each from 1 to 64 µg/mL, and amphotericin from 0.25 to 16 µg/mL. All plates had a drug‐free growth control well and a sterility control well.

For three‐dimensional microplate preparation, nine microplates were needed per isolate with each plate containing a fixed concentration of amphotericin in a twofold series, ranging from 0 to 8 µg/mL. The concentration of tamoxifen ranged from 0.25 to 64 µg/mL on the *x*‐axis, and the concentration of fluconazole varied from 1 to 32 µg/mL on the *y*‐axis. As before, 50 µL of each drug was used at fourfold the desired final concentration, but only 50 µL of inoculum was used per well, at a higher density of 1.0‐5.0 × 10^3^ CFU/mL, to deliver a final reaction volume of 200 µL. Every plate included a drug‐free growth control well and a sterility control well. The MIC of a drug combination was defined as the lowest concentration for which no growth was observed. Results were recorded 72 hours after incubation at 35°C, and all plates were assessed by two observers.

### 2.4 Statistical analysis

Susceptibility was expressed as the minimum inhibitory concentration (MIC) of the particular drug at which growth was inhibited for 50% (MIC_50_), and 90% (MIC_90_) of all isolates, and as the geometric mean inhibitory concentration of each drug. The differences in MICs of amphotericin and fluconazole when incubated with and without tamoxifen were compared using the Wilcoxon rank‐sum test.

Evidence of drug interaction was evaluated using the fractional inhibitory concentration index (FICI). The FICI is the sum of the fractional inhibitory concentrations (FIC) of each drug in the 2‐ or 3‐dimensional testing. The FIC is calculated by dividing the MIC of the drug when used in combination by the MIC of that same drug when used alone. For two‐dimensional chequerboard testing, the FICI is interpreted as follows: FICI ≤ 0.5 = evidence of synergy; 0.5 < FICI ≤ 4 = no evidence of drug interaction; FICI > 4 = evidence of drug antagonism.^20^ For three‐dimensional chequerboard testing, an FICI of <1.0 is considered evidence of synergy, and an FICI of greater than one is defined as antagonism; an FICI equal to one is considered evidence of no interaction.^21^

All analyses were done using r software version 3.1.2.^22^

### 2.5 Ethics

The authors confirm that the ethical policies of the journal, as noted on the journal's author guidelines page, have been adhered to. All clinical studies from which the isolates were derived had ethical approval from the Hospital for Tropical Diseases, and either Liverpool School of Tropical Medicine, UK or the Oxford Tropical Ethics Committee, UK. All participants gave informed consent.

## 3 RESULTS

The drug susceptibility (MIC_50_, MIC_90, _ranges and geometric means) by species are summarised in Table 1. Tamoxifen MICs ranged from 2 to 16 µg/mL, with an MIC_50_ of 4 µg/mL and MIC_90_ of 16 µg/mL. While clinical breakpoints are not defined for *C. neoformans*, a range of susceptibilities to fluconazole and flucytosine were seen among the isolates, including nine isolates which could be considered to have dose‐dependent susceptibility or frank resistance to fluconazole (MIC ≥ 16 µg/mL; three where the MIC = 64 µg/mL) and 14 isolates with less than full susceptibility to flucytosine (MIC > 4µg/mL; eight where the MIC ≥ 16 µg/mL).^10^, ^23^, ^24^ There was little variability in amphotericin B susceptibility among the clinical isolates. The MICs of amphotericin B, tamoxifen, fluconazole and flucytosine were 1, 4, 4 and 4 µg/mL, respectively, for the type strain H99. *C. gattii* species complex strains had similar susceptibilities to tamoxifen as *C. neoformans*, ranging from 2 to 8 µg/mL, with no clear difference by species (Table 1).

**Table 1:** In vitro susceptibility of Vietnamese clinical isolates of *Cryptococcus neoformans* and *Cryptococcus gattii* isolates to tamoxifen, amphotericin, fluconazole and flucytosine

Antifungal (No tested)	Minimum inhibitory concentration (MIC, µg/mL)			
	Range	MIC50	MIC90	Geometric Mean
*C. neoformans* a				
Tamoxifen (30)	2‐16	4	16	7.1
Amphotericin B (30)	0.25‐2	1	2	1.1
Fluconazole (20)	0.5‐64	8	64	9.2
Flucytosine (20)	4‐32	8	16	8.9
*C. gattii sensu lato* b				
Tamoxifen (8)	2‐8	4	8	4
Amphotericin B (8)	0.25‐2	0.5	1	0.6

a Excludes results for H99. Numbers in brackets are number of isolates tested.

b Includes data from four Vietnamese clinical isolates (three *C. gattii sensu stricto* and one *C. deuterogatii*, and the four control strains).

### 3.1 The combination of tamoxifen with amphotericin B, but not with fluconazole or flucytosine, appears synergistic for the majority of clinical isolates of *C. neoformans*

The results of chequerboard testing are shown in Table 2. The combination of tamoxifen with amphotericin suggested a synergistic interaction for 20 of 30 isolates (67%) synergy. However, there was evidence of synergy in only one of 20 isolates (5%) when tamoxifen was combined with fluconazole, and none when combined with flucytosine. There was no evidence of antagonism between any of the tamoxifen‐antifungal combinations, suggesting that the effects of fluconazole or flucytosine and tamoxifen are additive for most strains.

**Table 2:** Evidence of drug interactions from two‐dimensional chequerboard testing of tamoxifen in combination with either amphotericin, fluconazole or flucytosine

Antifungal combination	Proportion (%) of isolates where particular drug interactions was observed^a^		
	Synergy FICI ≤ 0.5	No interaction 0.5 < FICI ≤ 4	Antagonism FIC > 4
*Cryptococcus neoformans*			
Tamoxifen + amphotericin	67 (20/30)	33 (10/30)	0 (0/30)
Tamoxifen + fluconazole	5 (1/20)	95 (19/20)	0 (0/20)
Tamoxifen + flucytosine	0 (0/20)	100 (20/20)	0 (0/20)
*Cryptococcus gattii sensu lato* b			
Tamoxifen + amphotericin	75% (6/8)	25% (2/8)	0 (0/8)

a Numbers in brackets: Numerators are the numbers of strains where interaction was observed; denominators are the numbers of isolates tested.

b Includes data from four Vietnamese clinical isolates (three *C. gattii sensu stricto* and one *C. deuterogatii*, and the four control strains).

Similarly, there was apparent synergy between tamoxifen and amphotericin for six of eight *C. gattii* isolates (75%, three of four Vietnamese clinical strains and three of four control strains) (Table 3). Interestingly, the two strains where the drug combination appeared to be only additive were both *C. deuterogattii* (BMD800 and control strain WM178).

**Table 3:** Susceptibilities of individual strains of *Cryptococcus neoformans* and *Cryptococcus gattii* to amphotericin B and tamoxifen, alone and in combination

Isolate ID	MIC (µg/mL)				FIC index
	Amphotericin B		Tamoxifen		
	Alone	Combined	Alone	Combined	
BK03	2	0.5	8	2	0.5
BK20	0.5	0.25	4	2	1
BK23	1	0.25	4	0.5	0.38
BK33	1	0.25	16	4	0.5
BK34	2	0.125	4	4	1.06
BK42	0.5	0.25	4	0.5	0.63
BK45	1	0.25	4	1	0.5
BK48	0.5	0.125	4	1	0.5
BK59	1	0.5	8	2	0.75
BK62	0.5	0.125	4	1	0.5
BK69	2	0.25	2	2	1.13
BK73	2	0.5	16	0.25	0.27
BK81	1	0.25	16	2	0.38
BK84	1	0.25	16	2	0.38
BK87	2	0.5	4	0.25	0.31
BK91	2	0.5	16	0.5	0.28
BK111	1	0.25	4	1	0.5
BK115	0.5	0.25	2	1	1
BK128	2	0.25	8	2	0.38
BK139	1	0.25	4	1	0.5
BK169	0.5	0.25	4	1	0.75
BK175	1	0.25	4	0.25	0.31
BK192	2	0.125	16	4	0.31
BK224	0.25	0.125	4	2	1
BK247	2	0.25	16	2	0.25
BK287	1	0.25	8	2	0.5
BK301	2	1	16	1	0.56
BMD394	2	0.5	4	0.25	0.31
BMD865	1	0.125	4	0.5	0.25
BMD1392	2	0.125	4	4	1.06
BMD800^a^	1	0.125	8	4	0.63
BMD856^b^	2	0.5	2	0.25	0.38
BMD1377^b^	0.5	0.125	2	0.5	0.50
BMD1516^b^	0.5	0.125	4	0.25	0.31
WM179	0.5	0.125	4	0.25	0.31
WM178	0.25	0.125	4	0.25	0.56
WM175	1	0.25	8	0.25	0.28
WM779	0.5	0.125	4	0.25	0.31
H99	1	0.25	4	2	0.8

a *C. deuterogattii*.

b *C. gattii sensu stricto.*

When tamoxifen and amphotericin were combined, there was a statistically significant reduction in the geometric mean MIC of amphotericin B from 1.1 to 0.26 µg/mL (difference between the groups *P* < 0.001) (Table 3) for the 30 *C. neoformans* isolates. While there was little evidence of a synergistic interaction between tamoxifen and fluconazole, there was a decrease in the geometric mean MIC of fluconazole from 9.19 to 2.30 µg/mL when the two drugs were combined (difference between the groups *P* = 0.005, Table S1). This was also seen for the combination of tamoxifen and flucytosine where the geometric mean MIC of flucytosine fell from 8.9 to 1.2 µg/mL (monotherapy versus combination therapy, difference between the groups *P* < 0.001, Table S2).

Of isolates that appeared to have reduced susceptibility to fluconazole, four of the nine had at least fourfold increases in susceptibility when combined with tamoxifen. All 14 strains with probable reduced susceptibility to flucytosine had eightfold or greater increases in susceptibility to the drug (to 1 μg/mL) when combined with tamoxifen.

### 3.2 Synergy is preserved when tamoxifen, amphotericin and fluconazole are combined in triple combination

We tested the three drugs—tamoxifen, amphotericin and fluconazole—in combination against four clinical *C. neoformans* isolates and the H99 type strain. The amphotericin‐tamoxifen dual drug combination had appeared synergistic with two of the selected isolates (FICI = 0.5), but to have only additive effect with the other two (0.5 < FICI ≤ 4). In the triple combination testing (Table 4), there appeared to be a synergistic drug interaction for three of the four clinical isolates (FICI < 1), no evidence of antagonism for the remaining clinical isolate (FIC = 1) and a suggestion of antagonism for the H99 type strain (FICI > 1).

**Table 4:** Minimum inhibitory concentration of Amphotericin B, Fluconazole and Tamoxifen, in triple combination, against *Cryptococcus neoformans*

Isolate ID	MIC (µg/mL)						FIC index
	Amphotericin B		Fluconazole		Tamoxifen		
	Alone	Combined^a^	Alone	Combined^a^	Alone	Combined^a^	
BK03	2	0.5	16	1	4	1	0.56
BK59	1	0.25	8	1	2	1	0.88
BK287	2	0.125	8	1	4	2	0.69
BK301	2	1	4	1	2	0.5	1
H99	0.5	0.0625	2	2	2	0.25	1.3

a The combined result describes the MIC of that drug when combined with the two other antifungal drugs.

## 4 DISCUSSION

Cryptococcal meningitis, even with gold standard therapy, is a devastating illness with mortality rates in the order of 30% 10 weeks after diagnosis.^1^, ^6^, ^9^ The best outcomes depend on induction treatment with amphotericin and flucytosine over the first 1‐2 weeks.^4^, ^6^, ^9^ Unfortunately, flucytosine is usually neither available nor affordable where the major burden of disease occurs; flucytosine‐sparing therapies have significantly higher mortalities.^10^ There is a clear need to develop treatment that is more effective, tolerable, available and affordable. However, new drugs developed specifically to treat cryptococcal disease are unlikely to fulfil the last two of these criteria in low‐income settings.

Here, we have confirmed that tamoxifen, a selective oestrogen receptor modulator used most frequently to treat breast cancer, and which was shown to have an anti‐cryptococcal effect against the type strain almost 10 years ago, has in vitro activity against clinical isolates of *C. neoformans* and *C. gattii sl* in Vietnam. Tamoxifen has excellent bioavailability, with minimal first pass metabolism, but standard doses of 20‐60 mg/d, as used to treat breast cancer, achieve serum concentrations of only around 0.1 µg/mL.^25^, ^26^ This is clearly somewhat lower than the MIC_50_ and MIC_90_ of our isolates (4 and 16 µg/mL, respectively). However, considerably higher doses—240‐500 mg/d—have been studied in clinical trials for central nervous system tumours, desmoid tumours and lung cancer and are well‐tolerated.^27^, ^28^, ^29^, ^30^, ^31^, ^32^ Such treatment regimens achieve serum tamoxifen concentrations around 2‐8 µg/mL, approaching the MIC_50_ seen in our study, and have been administered safely for up to 1 year. Moreover, tamoxifen is concentrated in lipid‐rich tissues including the brain, the site of disease in cryptococcal meningitis.^17^ Here, concentrations are 40‐ to 100‐fold higher than in serum and thus likely to safely exceed the MIC in the majority of infections. Tamoxifen is also concentrated within macrophages, an important site of replication of *C. neoformans*.^15^ Therefore, it is likely that oral administration of tamoxifen can achieve sufficient concentrations of tamoxifen at the site of disease to have therapeutic effect.

Furthermore, we confirmed that tamoxifen appears to have a synergistic effect when combined with amphotericin, as has been reported previously for the H99 type strain.^15^ This was seen in the majority (67%) of our clinical isolates (and is similar to reported rates of synergy with amphotericin and flucytosine in clinical isolates^33^). Of note, we did not see evidence of synergy with *C. deuterogattii*, although only two strains were tested. However, in contrast with the previous report, we found the interaction between tamoxifen and fluconazole to be simply additive for almost all (95%) of our isolates. Importantly, there was no evidence of antagonism between tamoxifen and any of amphotericin, fluconazole or flucytosine. When we combined the three drugs, we found that a synergistic interaction was preserved in strains where it had been seen for the amphotericin‐tamoxifen dual combination. In addition, combining the three drugs also seemed to deliver a synergistic interaction in one of the clinical strains where synergy had not been apparent for the dual drug combination. Therefore, a triple drug combination has the potential to be synergistic in at least two‐thirds of Vietnamese cases. However, we also found that the combination of amphotericin, fluconazole and tamoxifen appeared antagonistic for the H99 type strain and were unable to replicate previously published data that tamoxifen was synergistic when combined with either amphotericin or tamoxifen for the type strain. The reasons for this are not clear. However, microevolution and divergence of the H99 type strain in different laboratories are well‐documented.^34^

Synergy is an attractive property for an anti‐microbial treatment, promising non‐linear gains in efficacy, potentially with little additional side effect cost. There has been recent interest in the use of sertraline to treat cryptococcal meningitis. Sertraline also appears to work synergistically with either amphotericin or fluconazole against *C. neoformans *in vitro.^35^, ^36^ A (historically) controlled phase 2 trial of this drug in combination with amphotericin and fluconazole appeared promising, suggesting improved rates of clearance of yeast from cerebrospinal fluid, although this contrasted with a smaller controlled trial which found no difference.^37^, ^38^ Disappointingly, a recent phase 3 randomised placebo‐controlled trial of sertraline boosted therapy, powered to survival, was stopped early due to futility after enrolling 460 patients.^39^ No differences were found between the sertraline or placebo arms in patient survival or yeast clearance rates from cerebrospinal fluid.

Despite the fact that we can discern a range of susceptibilities to tamoxifen in vitro, there is no clear relationship between antifungal susceptibility in vitro and response to treatment in human cryptococcal disease—clinical breakpoints are not defined and outside of relapsed disease the value of susceptibility testing is unclear.^7^, ^40^ However, the rate of clearance of yeast from cerebrospinal fluid—early fungicidal activity (EFA)—does appear to correlate with survival in cryptococcal meningitis at the population level.^5^, ^6^, ^9^ Therefore, as a next step, tamoxifen should be tested in a small randomised controlled trial, to generate initial efficacy (EFA) and safety data. A dose in the order of 300 mg/d would be needed to attain serum levels around the MIC_90_. While such a dose is likely to be less tolerable than that used in breast cancer, it must be considered in the context of the poor prognosis of cryptococcal meningitis, which compares unfavourably with glioblastoma, prostate and small cell lung cancer, where large doses of tamoxifen have been used.^41^ Recognised side effects include thromboembolic disease and retinopathy (although the risk of these is low with short duration treatment) and QT prolongation of the cardiac cycle.^42^ QT prolongation might be expected to be a particular risk as doses are increased, especially when combined with fluconazole, which can also cause this conduction abnormality, and disordered serum electrolytes, frequently seen in cryptococcal disease as a consequence of amphotericin therapy and intracranial pathology.^43^, ^44^, ^45^ Any study would need to pay close attention to these risks, and a trial is underway in Vietnam (Clinicaltrials.gov NCT03112031).

## CONFLICTS OF INTEREST

None to declare. The funders had no role in study design, analysis or decision to publish.

## AUTHOR CONTRIBUTIONS

J.N.D., N.T.T.N. and D.K. conceived the study; T.P.H. and A.D.V. performed the laboratory work, T.P.H., L.T.H.N. and J.N.D. performed the analyses, N.P.H.L. and N.V.V.C. provided material support, T.P.H. prepared the first manuscript draft, all authors reviewed and revised the manuscript.
