# Supplementary material for: The combination of tamoxifen with amphotericin B, but not with fluconazole, has synergistic activity against the majority of clinical isolates of Cryptococcus neoformans
Source: Mycoses. 2019 Jun 23;62(9):818–25. doi: 10.1111/myc.12955 (PMC6771715; doi:10.1111/myc.12955)
Supplement: Supplementary file 1 [file MYC-62-818-s001.docx]

**Table S1. In vitro activity of fluconazole and tamoxifen, alone and combination, against *C. neoformans.***

| Isolate | Minimum Inhibitory Concentration (mg/L) | | | | FICI* |
| --- | --- | --- | --- | --- | --- |
|  | **Fluconazole** | | **Tamoxifen** | |  |
|  | Alone | Combined | Alone | Combined |  |
| BK03 | 64 | 32 | 8 | 8 | **1.5** |
| BK23 | 2 | 0.5 | 8 | 4 | **0.75** |
| BK33 | 16 | 16 | 16 | 16 | **2.0** |
| BK34 | 0.5 | 0.5 | 4 | 1 | **1.25** |
| BK48 | 2 | 2 | 8 | 4 | **1.5** |
| BK59 | 16 | 1 | 2 | 2 | **1.1** |
| BK69 | 8 | 1 | 4 | 2 | **0.6** |
| BK81 | 64 | 32 | 16 | 16 | **1.5** |
| BK84 | 16 | 1 | 2 | 2 | **1.1** |
| BK91 | 32 | 8 | 2 | 2 | **1.3** |
| BK111 | 8 | 1 | 8 | 4 | **0.6** |
| BK115 | 2 | 0.5 | 4 | 4 | **1.25** |
| BK128 | 8 | 1 | 2 | 1 | **0.6** |
| BK139 | 8 | 1 | 8 | 4 | **0.6** |
| BK175 | 4 | 2 | 8 | 4 | **1** |
| BK192 | 4 | 0.5 | 8 | 8 | **1.125** |
| BK224 | 4 | 1 | 8 | 2 | **0.5** |
| BK247 | 32 | 1 | 2 | 2 | **1.0** |
| BK287 | 64 | 32 | 16 | 16 | **1.5** |
| BK301 | 16 | 16 | 8 | 8 | **2.0** |
| H99 | 4 | 1 | 2 | 1 | **0.8** |
| **FICI = fractional inhibitory concentration index. An FICI ≤0.5 is considered evidence of synergy; 0.5 <FICI ≤ 4.0 – no interaction; FIC> 4.0 – antagonism.* | | | | | |

**Table S2. In vitro activity of flucytosine and tamoxifen, alone and in combination, against *C. neoformans.***

| Isolate | Minimum Inhibitory Concentration (mg/L) | | | | FICI* |
| --- | --- | --- | --- | --- | --- |
|  | **Flucytosine** | | **Tamoxifen** | |  |
|  | Alone | Combined | Alone | Combined |  |
| BK03 | 8 | 1 | 2 | 2 | **1.1** |
| BK23 | 4 | 4 | 8 | 4 | **1.5** |
| BK033 | 8 | 1 | 2 | 2 | **1.1** |
| BK034 | 4 | 1 | 4 | 2 | **0.75** |
| BK48 | 4 | 4 | 8 | 4 | **1.5** |
| BK059 | 8 | 1 | 4 | 4 | **1.1** |
| BK69 | 4 | 1 | 8 | 8 | **1.25** |
| BK081 | 16 | 1 | 2 | 2 | **1.1** |
| BK84 | 16 | 1 | 2 | 2 | **1.1** |
| BK91 | 16 | 1 | 4 | 4 | **1.1** |
| BK111 | 32 | 1 | 4 | 4 | **1.0** |
| BK115 | 4 | 1 | 4 | 4 | **1.25** |
| BK128 | 16 | 1 | 2 | 2 | **1.1** |
| BK139 | 16 | 1 | 2 | 2 | **1.1** |
| BK175 | 4 | 4 | 8 | 4 | **1.5** |
| BK192 | 8 | 1 | 4 | 4 | **1.1** |
| BK224 | 16 | 1 | 2 | 2 | **1.1** |
| BK247 | 8 | 1 | 2 | 2 | **1.1** |
| BK287 | 8 | 1 | 2 | 2 | **1.1** |
| BK301 | 16 | 1 | 2 | 2 | **1.1** |
| H99 | 4 | 4 | 2 | 0.25 | **1.1** |
| **FICI = fractional inhibitory concentration index. An FICI ≤0.5 is considered evidence of synergy; 0.5 <FICI ≤ 4.0 – no interaction; FIC> 4.0 – antagonism.* | | | | | |
